# The Meaning of Diathesis

**Published:** 1910-10-22

**Authors:** 


The Hospital

A Journal of

The Medical Sciences and Hospital Administration.

New Series. No. 193, Vol. VIII. [No. 1265, Vol. XLIX.] SATURDAY, OCTOBER 22, 1910.

### The Meaning of Diathesis.

One of the intricate and fascinating problems of medical philosophy, and one which is still very far from being solved, is that connected with the idiosyncrasies of the "constitution." From time immemorial it has been supposed that certain individuals possess undue susceptibilities to certain diseases; and after the old humoral nosology was abandoned physicians were wont to speak of scrofulous, gouty, rheumatic, and other "diatheses." Some of these diatheses could, it was thought, be recognised by the physical attributes of their possessors; and to some degree this opinion is still held. Early last year attention was drawn in these columns to a novel view of diathesis, expounded by Dr. Leslie Mackenzie (The Hospital, January 16, 1909, p. 394). In a work on Skin Diseases,* quite recently translated into English, a French author enters afresh into this question, and produces yet another conception of it.

So many contending theories have been invented that it would be difficult to lay down exactly what the term diathesis conveys to the bulk of practitioners. Professor Gaucher, however, is in no sort of doubt what it means to him. He defines diathesis as "a chronic auto-intoxication by nitrogenous extractive matters"; and he expressly excludes auto-intoxications due to chronic renal disease and to abnormal fermentations in the alimentary canal, as in a dilated stomach. There are in some people, according to this view, "autogenous morbid poisons" which provoke alternately cutaneous eruptions and internal affections; and the subject of such an intoxication is held to exhibit "diathesis." Moreover, there is but one diathesis—not several, as the old school of physicians believed. To this diathesis he gives the name of "arthritism": it corresponds to the arthritic and herpetic diatheses of the ancients. There are included among the arthritics two types—fat arthritics, with a gouty tendency, and thin arthritics, who represent the old herpetics. The "diathetic dermatoses" arise from arthritism, and include eczema, pityriasis simplex, psoriasis, prurigo, the different forms of lichen, acne rosacea, etc. The fundamental character of arthritism is a disturbance of nutritive changes, an impairment of nutrition, on account of which the substances absorbed and assimilated undergo incomplete oxidation. Scrofula is not recognised as having any claim to be regarded as a diathesis. Scrofulides are mostly forms of tuberculosis; and the remainder are due to hereditary syphilis, or to special microbic infections. They may, however, be superadded to a skin disease which is diathetic by origin and thus produce hybrids —"diathetic cutaneous affections which imprint a special stamp on the soil in which they develop."

The old doctrine of *metastasis* is also made part and parcel of this conception of diathesis as the fundamental cause of the majority of cutaneous diseases. By this was meant, in dermatology, the replacement of a benign cutaneous manifestation of the diathesis by a visceral determination more grave, sometimes even fatal. Thus the disappearance of generalised eczema has been known a good many times to coincide with the development of asthma, rheumatism, bronchitis, or enteritis; prurigo may alternate with bronchitis, psoriasis with rheumatism. The skin, says Professor Gaucher, is an emunctory for the morbid poisons elaborated in the metabolism of the arthritic; the dermatosis is a safeguard, and when it disappears the visceral manifestations follow, because the toxins accumulate in the internal organs instead of being eliminated through the skin, which they irritate in the process. The conception of the skin as a safety-valve for the excretion of the humours which may otherwise cause various diseases is one with which the general public is both familiar and sympathetic. But in the entire absence of any proof that the varied symptoms and diseases which are here ascribed to "arthritism" have anything whatever to do with insufficient oxidation of nutritive materials, the medical profession may well be somewhat sceptical as to the correctness of these very sweeping generalisations.

Take, for example, the case of erythema multiforme, including erythema nodosum. Many physicians hold that this rash is especially associated with acute rheumatism; that is, that it occurs in those who have a personal or family history of that disease. Now, rheumatism is, we all believe, a specific disease, and in this country most medical men credit Drs. Poynton and Paine with having discovered the specific micro-organism. On the other hand, a number of observers of late years have thrown doubt on the rheumatic origin of erythema, and have identified it as a tuberculide. Both schools have this in common, that they ascribe it to the presence of a toxin of microbic origin, not to an endogenous auto-intoxication whose other manifestations include the majority of skin diseases, as well as such varied disorders as gout, gall-stones, dyspepsia, arterio-sclerosis, and bronchitis. Nobody has yet recorded any unusual incidence of erythema in dyspeptics or bronchitics, nor indeed in anything except rheumatism and tuberculosis, and doubtfully in those. Professor Gaucher's theory explains too much; it is too vague in details, and throws so very little light into the dark corners of pathology. His "sort of constitutional condition, a general disposition of the organism," is an ill for which he suggests no remedy. Even if it were proved true, it yet avails nothing for any practical purpose; and, in fact, there is no real attempt to prove that it is true. As a speculation—the outcome of that Gallic facility which in some ways the English can but admire without being able to imitate—it is highly interesting. But as a contribution to science it cannot be regarded as likely to make headway without a great deal more evidence than is offered in Professor Gaucher's text-book.

* *Diseases of the Skin.* By E. Gaucher. Translated and edited by C. F. Marshall. London: John Murray. 1910.

